# Supplementary material for: Sex and stress interactions in fear synchrony of mouse dyads
Source: bioRxiv. 2024 Jun 10:2024.06.09.598132. Preprint. [Version 1] doi: 10.1101/2024.06.09.598132 (PMC11195068; doi:10.1101/2024.06.09.598132)
Supplement: Supplement 1 [file NIHPP2024.06.09.598132v1-supplement-1.pdf]

bioRxiv preprint doi: https://doi.org/10.1101/2024.06.09.598132; this version posted June 10, 2024. The copyright holder for this preprint (which was not certified by peer review) is the author/funder, who has granted bioRxiv a license to display the preprint in perpetuity. It is made available under aCC-BY 4.0 International license.

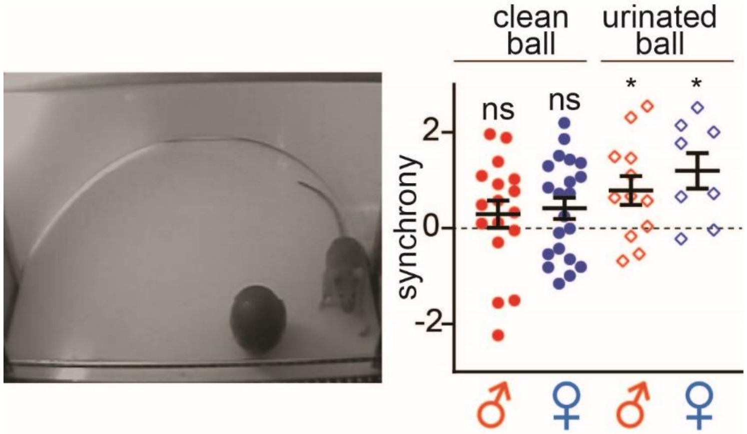

Supplementary Fig1. Mice do not synchronize with non-social ball, but urine-painting on the ball enables synchrony. Left Synchrony test with non-social ball. **Right** Summary of synchrony for each group (clean ball: n=17 (male), 21 (female), urinated ball: n=12 (male), 8 (female)). One-sample t-test comparing to 0: * $p<0.05$.

bioRxiv preprint doi: https://doi.org/10.1101/2024.06.09.598132; this version posted June 10, 2024. The copyright holder for this preprint (which was not certified by peer review) is the author/funder, who has granted bioRxiv a license to display the preprint in perpetuity. It is made available under aCC-BY 4.0 International license.

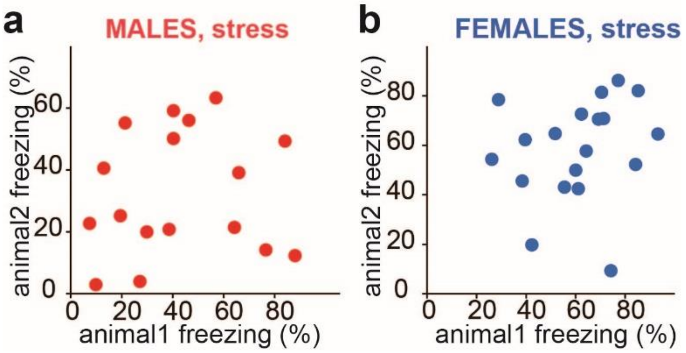

Supplementary Fig2. Freezing levels of stressed partners do not correlate. Scatter plots of partners' freezing levels in male (**a**) (n=17) and female (**b**) (n=19) dyads.

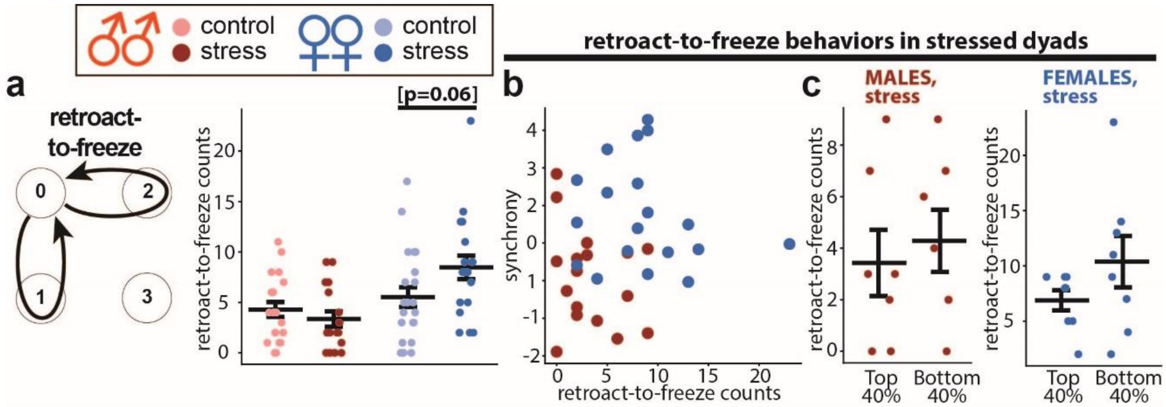

Supplementary Fig3. Stress effects on retroact-to-freeze. a "Retroact-to-freeze" scheme (**left**) and counts in control and stressed dyads (**right**). **b** Scatter plot between synchrony and "retroact-to-freeze" counts in stressed dyads. **c** Comparisons of "Retroact-to-freeze" counts between the top and bottom 40% of stressed dyads ranked by synchrony. Colors representing sex (red: male dyads, blue: female dyads) and treatments (light symbols: control, males: n=20, females: n=23, dark symbols: stress, males: n=17, females: n=19). Horizontal bars on diagrams indicate means±SEM.

bioRxiv preprint doi: https://doi.org/10.1101/2024.06.09.598132; this version posted June 10, 2024. The copyright holder for this preprint (which was not certified by peer review) is the author/funder, who has granted bioRxiv a license to display the preprint in perpetuity. It is made available under aCC-BY 4.0 International license.

Supplementary Table 1. The list of dyad groups in the study

Sex composition	Dyad type	Group size in dyad or singal animal	Figures	Appeared group name
same-sex dyads	familiar	20 (male dyad), 23 (female dyad)	Fig1, Fig2	"males", "females"
			Fig3, Sup Fig3	"control males", "control females"
			Fig5	"familiar males", "familiar females"
	familiar stressed	17 (male dyad), 19 (female dyad)	Fig3, Sup Fig2, Sup Fig3	"stress males", "stress females"
	unfamiliar	19 (male dyad), 20 (female dyad)	Fig5	"unfamiliar males", unfamiliar femaels"
	dmPFC infusion and stress	11 (male dyad, muscimol), 10 (male dyad, vehicle)	Fig4	"muscimol+stress", "vehicle+stress"
oposite-sex dyads	familiar	14 (dyad)	Fig6	"control"
	familiar stressed	14 (dyad)		"stress"
	unfamiliar	16 (dyad)		"unfamiliar"
single animals	with clean ball	17 (male), 21 (female)	Sup Fig1	"male with clean ball", "female with clean ball"
	with urinated ball	12 (male), 8 (female)		"male with clean ball", "female with clean ball"
